# A standardized extract of *Echinacea purpurea* containing higher chicoric acid content enhances immune function in murine macrophages and cyclophosphamide-induced immunosuppression mice

**DOI:** 10.1080/13880209.2023.2244000

**Published:** 2023-08-10

**Authors:** Heggar Venkataramana Sudeep, Kuluvar Gouthamchandra, Illuri Ramanaiah, Amritha Raj, Puttaswamy Naveen, Kodimule Shyamprasad

**Affiliations:** R&D Center for Excellence, Vidya Herbs Pvt Ltd, Bangalore, India

**Keywords:** Purple cone flower, immunity, herbal supplement, cytokines

## Abstract

**Context:**

Preparations of *Echinacea* have been used by herbalists to boost the immune system.

**Objective:**

In this study, *Echinacea purpurea* (L.) Moench (Asteraceae) extract with enriched chicoric acid content was investigated for immunomodulation.

**Materials and methods:**

The standardized hydroalcoholic extract (4% chicoric acid) was prepared from the aerial parts of *E. purpurea* (SEP). The extract was screened for *in vitro* antioxidant activities, and immunomodulation in RAW 264.7 cells, at 200 and 400 µg/mL. Further, the male BALB/c mice (20-25 g) were divided into 4 groups (*n* = 6 per group). All the groups except control, were intraperitoneally injected with 70 mg/kg/day of cyclophosphamide (CTX) for 4 consecutive days. The treatment groups received SEP extract (100 and 200 mg/kg body weight) *p.o.* from day 5 to 14.

**Results:**

The SEP extract inhibited DPPH (IC50 = 106.7 µg/mL), ABTS^+^ (IC_50_ = 19.88 µg/mL) and nitric oxide (IC_50_ = 120.1 µg/mL). The SEP extract’s ORAC (oxygen radical absorbance capacity) value was 1931.63 µM TE/g. In RAW 264.7 cells, SEP extract increased the nitric oxide production by 30.76- and 39.07-fold at 200 and 400 µg/mL, respectively, compared to the untreated cells. SEP extract significantly increased phagocytosis and cytokine release (TNF-α, IL-6, and IL-1β) in the cells. Further, the extract improved immune organ indices, lymphocyte proliferation and serum cytokine levels in CTX-induced mice. The extract at 200 mg/kg significantly increased the natural killer cell activity (24.6%) and phagocytic index (28.03%) of CTX mice.

**Conclusion:**

Our results strongly support SEP extract with 4% chicoric acid as a functional ingredient for immunomodulation.

## Introduction

The term immunity defines the body’s natural defense system against a massive array of diseases and disorders. Surprisingly complicated and advanced among vertebrates, the complex immune system is capable to produce a boundless variety of cells and molecules to block enormous variety of infections and undesirable elements (Patil et al. 2012; Dutt 2013; Sharma et al. 2017). The strategy of avoiding them all may be extraordinarily tricky or difficult and many of the pathogens have mechanisms that allow them to evade the full effects of host defenses. The innate immune responses are generally rapid and independent of immunological memory unlike the adaptive immunity. Innate immune responses include the cellular mechanisms, for example phagocytosis and cytotoxicity, or secretions (cytokines, complimentary factors, antimicrobial peptides, etc.) (Degn and Thiel 2013; Hilchie et al. 2013; Sokol and Luster 2015). Our knowledge of immune functioning is constantly evolving over the years. There exists a paradigm from emerging evidence that the innate and adaptive immunity have mutually interacted and overlapped at times (Gasteiger et al. 2017). With recent advances in the understanding of how cells communicate with each other to signal effector functions, it has become possible to conceive of strategies to manipulate these signaling pathways to influence host responses (Tzianabos 2000). Compounds that can interact with the immune system to upregulate or downregulate specific aspects of the host response can be classified as immunomodulators or biologic response modifiers.

Numerous natural products, including essential oils, have been evaluated for immunomodulatory activity. The *Echinacea* genus (Asteraceae) commonly known as cone flowers, has nine flowering plant species including *E. purpurea* (L.) Moench. Native to eastern and central North America, *E. purpurea* (purple cone flower) has been used since ancient times as a remedy for respiratory infections and inflammation (Percival 2000). *Echinacea* is a well-known immunomodulator also having anti-inflammatory and antimicrobial effects (Vetvicka and Vetvickova 2014; Karsch-Völk et al. 2015; Rondanelli et al. 2018). The modulatory effects of *Echinacea* on immune function are well-studied. *Echinacea* extracts demonstrated immunostimulatory activity by enhancing the phagocytic index in macrophages, cytokine production and natural killer (NK) cell activity in animals (Burns et al. 2010; Zhang et al. 2011).

The principal active constituents in *E. purpurea* are polysaccharides, alkamides, caffeic acid derivatives (chicoric acid, chlorogenic acid, echinacoside, cafteric acid, and cynarin) and glycoproteins (Pellati et al. 2004). Chicoric acid is one of the key bioactive constituents in *E. purpurea* attributing to its antioxidant and immunostimulatory effects (Thygesen et al. 2007; Zolgharnein et al. 2010). The content of chicoric acid may vary due to climatic conditions and cultivation methods, resulting in variable potential of the plant. In general, the chicoric acid in *E. purpurea* is about 1.5% (Zolgharnein et al. 2010). Here, we have prepared a standardized *E. purpurea* extract containing higher content of chicoric acid and investigated the potential antioxidant and immunostimulatory function in experimental models.

## Materials and methods

### Reagents

Chicoric acid (97%), 2,2-diphenyl-1-picrylhydrazyl (DPPH), sodium nitroprusside dihydrate were purchased from Himedia. 2,2-Azinobis(3-ethylbenzothiazoline-6-sulphonic acid (ABTS), *N*-(1-naphthyl) ethylenediamine dihydrochloride, potassium persulfate, fluorescein, azobis dihydrochloride (AAPH), trolox, cyclophosphamide, 3-(4,5-dimethylthiazol-2-yl)-2,5-diphenyl tetrazolium bromide (MTT), lipopolysaccharide (LPS) (*Escherichia coli* 0111: B4), Concanavalin A (ConA), sodium nitrite, Greiss reagent and dimethylsulphoxide were procured from Sigma Aldrich Co. Dulbecco’s modified eagle medium (DMEM), Fetal bovine serum (FBS), RPMI-1640 medium and penicillin-streptomycin solution were purchased from Gibco-BRL^®^ (Grand Island, NY). The enzyme linked immunosorbent assay (ELISA) kits were provided by Krishgen Biosystems, India.

### *Preparation of standardized* E. purpurea *(SEP) extract*

The aerial parts of *E. purpurea* (Himalayan origin) were collected during the month of October 2019. The plant material was identified and authenticated by Dr. Vinayaka, Mangalore University, Karnataka, India. The voucher specimen was stored at Biomedicinal Research Laboratory, Vidya Herbs Pvt Ltd., Bangalore, India (VH/20/EP/02). The powdered raw material (100 g) was extracted with 800 mL of 70% ethyl alcohol at 65–70 °C for about 3 h. The mixture was then cooled to room temperature and filtered. The extraction was repeated two more times with 600 mL of 70% ethyl alcohol. The pooled filtrate was concentrated to dryness using rotary evaporator under reduced pressure. The resultant extract was further dissolved in 500 mL of demineralized water and charged into adsorption column containing amberlite XAD-16 resin (resin volume was 300 mL). The resin column was washed thrice with 300 mL of water and finally eluted with 1 L of 80% ethyl alcohol. The collected eluent was concentrated under vacuum to dryness to yield 3–5 g of extract.

### High performance liquid chromatography (HPLC) analysis of chicoric acid

SEP extract was dissolved in methanol at 1 mg/mL concentration; filtered through 0.2 µ nylon syringe filter and subjected to HPLC analysis. Quantification of chicoric acid was performed on a Shimadzu LC2030 C Prominence-i (Japan) system using Kinetex XBC-18 column (100 Å, 150 mm × 4.6 mm, 5 μm pore size). Reverse phase elution was performed with 50 mM sodium acetate as solvent A and acetonitrile as solvent B with flow rate of 0.5 mL/min and the injection volume of 10 µL. The column temperature was maintained at 28 °C, and the UV detector set at 335 nm. The gradient elution composed of 10–90% of solvent B at 0.01 to 1 min, followed by 90% of solvent B from 1 to 4 min and return to initial condition from 4 to 8 min, 10% of solvent B continued up to 10 min. The Chicoric acid content in the extract were calculated using the formula,

$$\%\text{ Chicoric acid}=\left({\text{Peak area}}_{\text{sample}}\times \text{concentration of standard }\times \text{ purity of standard}\right)/\left({\text{Peak area}}_{\text{standard}}\times \text{ concentration of sample}\right)$$

### In vitro studies

#### Determination of antioxidant activity

The antioxidant potential of SEP extract (2.5–300 µg/mL) was determined using *in vitro* assays: DPPH radical scavenging (Blois 1958), nitric oxide scavenging (Marcocci et al. 1994) and ABTS radical scavenging (Re et al. 1999) assays as described elsewhere.

Oxygen radical absorbance capacity (ORAC) assay was performed in accordance with Lucas-Abellán et al. (2008) with modifications. The assay was carried out on a Tecan Infinite 200 pro plate reader using Greiner black flat bottomed 96 well plate (chimney) with an excitation wavelength of 485 nm and emission wavelength of 525 nm. The reaction mixture contained 75 mM sodium phosphate buffer (pH 7.4) with 25 µL sample or buffer (for blank wells), fluorescein (100 µL, 3 nM final concentration). The reaction mixture was preincubated for 30 min at 37 °C followed by addition of freshly prepared AAPH solution (30 µL, 19 mM final concentration). Fluorescence was measured per minute for 1 h at 37 °C. The net area under the curve (AUC) is calculated using the data. Regression equation between Trolox concentration and net AUC was used to calculate the ORAC values of samples and are expressed as µM Trolox Equivalence/g.

#### Cell culture

The murine macrophage cell line RAW 264.7 was procured from National Center for Cell Sciences (NCCS), Pune, India. The cells were cultured in DMEM containing 10% FBS, 100 µg/mL streptomycin and 100 units/mL penicillin. The cells were maintained in a CO_2_ incubator at 37 °C.

#### MTT assay

Cytotoxicity of SEP extract was determined by the MTT assay as described (Mosaddegh et al. 2012). RAW 264.7 cells at a density of 1 × 10^4^ cells/well were seeded into a flat bottomed 96-well plate and incubated for 24 h at 37 °C in 5% CO_2_ incubator. Various concentrations (0.1–0.5 mg/mL) of SEP extract in the medium were discretely added to the plate wells. After 24 h, the supernatants were removed and 100 µL of MTT solution [5 mg/mL in phosphate buffered saline (PBS)] added to each well and further incubated for 4 h. The reduction of MTT was quantitated by measurement of the absorbance at 490 nm on the microplate reader (Tecan Infinite 200 Pro).

#### Measurement of nitric oxide (NO) production in RAW 264.7 macrophages

The cells (1 × 10^5^ cells/well seeded on a 96-well plate) were treated with SEP extract (200 & 400 μg/mL) and LPS (1 μg/mL) for 24 h. Later, the culture supernatants were incubated with same volume of Griess reagent for 15 min at room temperature. The absorbance was determined at 540 nm using microplate reader (Tecan Infinite 200 Pro). The calculated nitric oxide concentration was based on a nitrite calibration curve (1–100 μM).

#### Determination of phagocytosis

Effects of SEP extract on the phagocytosis of RAW264.7 cells were determined by using neutral red uptake method as described by previous literature (Luo et al. 2017). Briefly, the cells (10^5^ cells/mL) were seeded in 6-well plate and incubated for 24 h. Later, the cells were treated with two different concentrations of SEP extract (200 and 400 μg/mL). After 24 h of incubation, the supernatant was replaced with 1 mL of 0.1% neutral red solution, consequently incubated for 4 h. The cells were washed by using 0.1 M PBS for three times to remove excess dye solution. Finally, 100 μL of cell lysis solution (ethanol/acetic acid, 1:1, v/v) was added to each well. Thereupon, cell lysis solution was transferred to 96 well plate and the absorbance was measured at 540 nm by a microplate reader (Tecan Infinite 200 Pro).

#### Determination of levels of TNF-α, IL-6, and IL-1β

RAW264.7 cells (10^5^ cells/mL) were seeded in a 96-well plate and mixed with different dosages of SEP extract (200 and 400 μg/mL). After incubating for 24 h, levels of TNF-α (KB2145) and IL-6 (KB2068) in the supernatant were measured by ELISA assay according to manufactures’ instructions. Briefly, 100 µL/well of samples were added to ELISA plate and incubated for 2 h at ambient temperature. Later, the plate was washed with wash buffer and added 100 µL/well of biotinylated detection antibody to incubate for 1 h at room temperature. The plate was washed thoroughly and incubated with diluted streptavidin-HRP solution for 1 h. Subsequently, the plates were incubated with TMB substrate in the dark for 15 min. The reaction was stopped, and absorbance read at 450 nm. Similarly, IL-1β (E-EL-M0037) was measured in the cell culture supernatants with slight modifications as per the manufacturer’s instructions.

### In vivo *studies*

#### Animals and ethics approval

Male BALB/c mice weighing 20–25 g were purchased from Biogen, Bangalore (Reg No. 971/PO/RcBiBt/S/06/CPCSEA). The animals were housed in air-conditioned rooms with controlled environmental conditions (temperature 22 ± 3 °C and humidity 30–70%) with a 12 h light/dark cycle. The animals were given water and food (Commercial rodent pellet diet) *ad libitum*. The animal experiment was performed after the approval from the Institute of Animal Ethics Committee (IAEC) of Vidya Herbs Pvt Ltd, Bangalore, India (VHPL/PCL/IAEC/06/2020).

#### Experimental design

Twenty-four male BALB/c mice after 7-day acclimatization, were randomized to four groups (*n* = 6): normal control group (G1), cyclophosphamide (CTX) model group (G2), SEP extract low dose (SEP100) (G3) and high dose groups (SEP200) (G4). All the animals except the control group, were intraperitoneally administered 70 mg/kg CTX from day 1 to 4 to induce immunosuppression. Mice in SEP100 and SEP200 treatment groups were given 10 mL/kg of respective doses of SEP extract 100 and 200 mg/kg body weight by oral gavage daily from day 5 to 14. The selection of doses for CTX and SEP extract was based on previous literature (Park et al. 2018; Zhou et al. 2018). The SEP extract was solubilized in normal saline solution for dosing the animals. The control group animals received 1 mL of normal saline solution by gavage. At the end of experiment, all animals were euthanized by overdose of gaseous isoflurane anesthesia. Blood was collected in heparinized tubes and centrifuged at 900 *g* for 10 min. The plasma samples were stored at −20 °C for ELISA.

#### Immune organ indices

The spleen and thymus from the animals were carefully excised and weighed immediately to determine the organ indices using the formula: Spleen/thymus index (mg/g) = organ weight (mg)/body weight (g).

#### Preparation of mouse splenocyte suspension

The spleen tissues were gently ground in sterile phosphate buffer saline (PBS) using a mortar and passed through the cell strainer to prepare single cell suspension. The suspension was centrifuged at 250 *g* for 10 min. The collected splenocytes were resuspended in erythrocyte lysis buffer for 10 min in ice to remove the red blood cells. Then the mixture was centrifuged at 250 *g* for 5 min. The cell pellets were washed twice with PBS and added into 5 mL of RPMI 1640 medium supplemented with 10% FBS and 1% Penicillin/streptomycin solution in a humidified 95% air and 5% CO_2_ atmosphere. The cell density was adjusted to 2 × 10^5^ cells/mL.

#### Proliferation of splenic lymphocytes

The isolated mice splenocytes were seeded on to 96-well plate and then LPS (10 µg/mL) or ConA (5 µg/mL) was added. After 4 h incubation of the plates at 37 °C in CO_2_ incubator, MTT solution (5 mg/mL) was added into each well. The plates were further incubated for 4 h at 37 °C and 5% CO_2_. The purple formazan crystals were dissolved in dimethyl sulphoxide (DMSO), and the optical density (OD) read at 570 nm in a microplate reader (Tecan Infinite 200 Pro). The results were presented as stimulation index (Stimulus OD/Control OD).

#### Measurement of splenic NK cell activity

NK cell activity was measured using the mouse splenocyte suspension as effector cells and YAC-1 cells (National Center for Cell Sciences, Pune, India) as target cells. The cells were seeded in 96-well plate at effector to target cell ratio of 50:1 and co-cultured for 5 h at 37 °C in CO_2_ incubator. Later, 20 µL of MTT solution was added and incubated further for 4 h. Then the supernatant was transferred to another 96-well plate and added DMSO to solubilize the formazan product. The absorbance was measured at 490 nm and the NK cell activity (%) was obtained using the formula,

% NK cell activity=[Target cell OD−(Effector−target cell OD−Effector cell OD)/Target cell OD]×100

#### Peritoneal macrophage phagocytosis

The phagocytic ability of peritoneal macrophages isolated from the experimental mice was examined using the neutral red uptake as described elsewhere (Weeks et al. 1987; Chen et al. 2010). The macrophages aseptically harvested from the peritoneal lavage were suspended in RPMI-1640 medium and seeded in 96-well plate at a density of 5 × 10^5^ cells/well. The cells were cultured in 5% CO_2_ humidified incubator at 37 °C for 24 h. Then, the cells were washed three times with PBS and 0.075% neutral red solution was added. Following 1 h incubation, excess dye was removed by washing the cells with PBS. The cells were then lysed using a cell lysis buffer containing 1% acetic acid and ethanol and the absorbance read at 540 nm by a microplate reader.

#### Determination of serum cytokines

The blood samples were centrifuged at 1000 *g* for 10 min and the supernatant collected. The levels of inflammatory mediators (TNFα, IL-6, IL-1β, and IFN-γ) in serum samples were measured using commercial ELISA kits as per manufacturer’s instructions.

#### Histopathological examination

The isolated thymus and spleen tissues were fixed with 10% buffered formaldehyde and embedded in paraffin. Sections 4 μm thick were cut in Microtome (Leica RM 2125, Leica Microsystems GmbH, Wetzlar, Germany) and stained with hematoxylin and eosin. The dehydrated sections were mounted for imaging.

### Statistical analysis

The data were analyzed by Tukey’s multiple comparison test using GraphPad Prism 9.0. *p* < 0.05 was considered statistically significant.

## Results

### HPLC analysis of SEP extract

**Figure 1.**
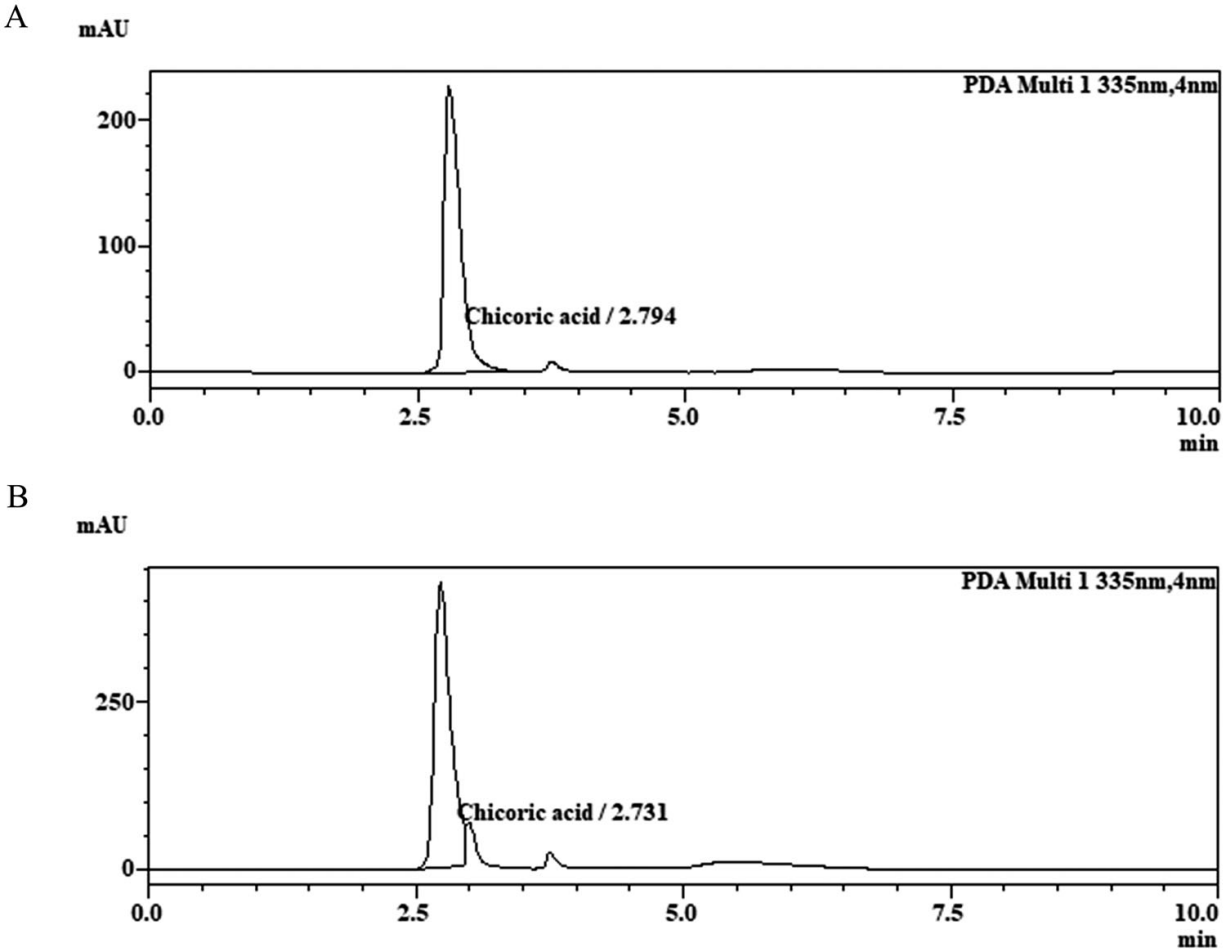
Optimized HPLC chromatograms of chicoric acid. (A) Reference standard chicoric acid, (B) SEP extract.

The quantitative analysis of SEP extract revealed the presence of 5.28% of chicoric acid. The retention time of standard and sample peak was found to be 2.794 min and 2.731 min respectively (Figure 1).

### In vitro free radical scavenging activity of SEP extract

**Figure 2.**
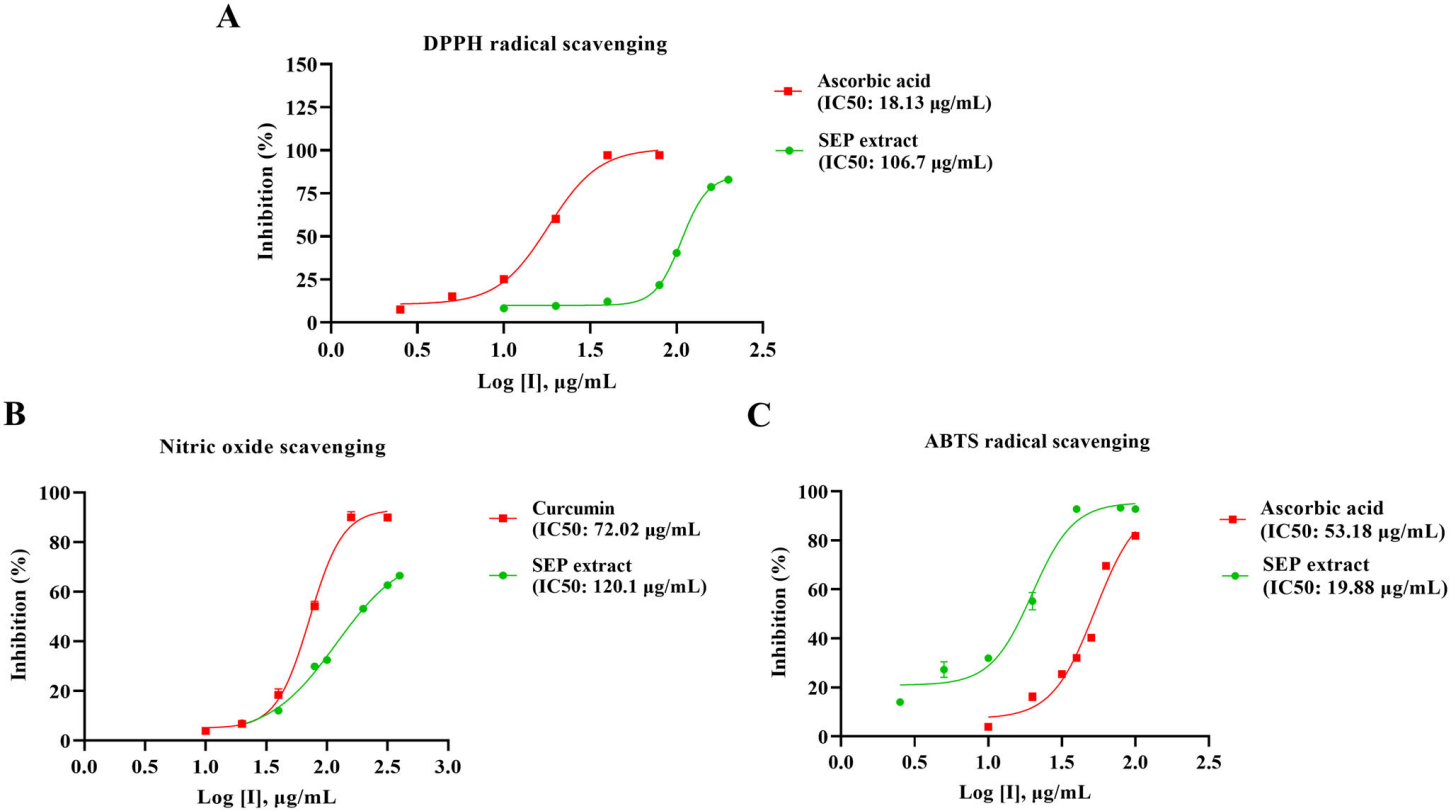
Determination of *in vitro* antioxidant activity of standardized *E. purpurea* (SEP) extract. (A) 2,2-diphenyl-1-picrylhydrazyl (DPPH) radical scavenging activity, (B) 2,2-azinobis-(3-ethylbenzothiazoline-6-sulfonate (ABTS^●+^) radical scavenging activity and (C) nitric oxide scavenging activity.

The free radical scavenging potential of SEP extract was assessed using *in vitro* assays. SEP extract exhibited concentration-dependent rise in the percentage inhibition of free radicals with the IC_50_ values of 106.7, 19.88, and 120.1 µg/mL, respectively, for DPPH, ABTS and nitric oxide assays. The reference compound ascorbic acid showed IC_50_ of 18.13 µg/mL and 53.18 µg/mL respectively for DPPH and ABTS radical scavenging. Curcumin showed marked inhibition of nitric oxide with an IC_50_ of 72.02 µg/mL (Figure 2).

**Figure 3.**
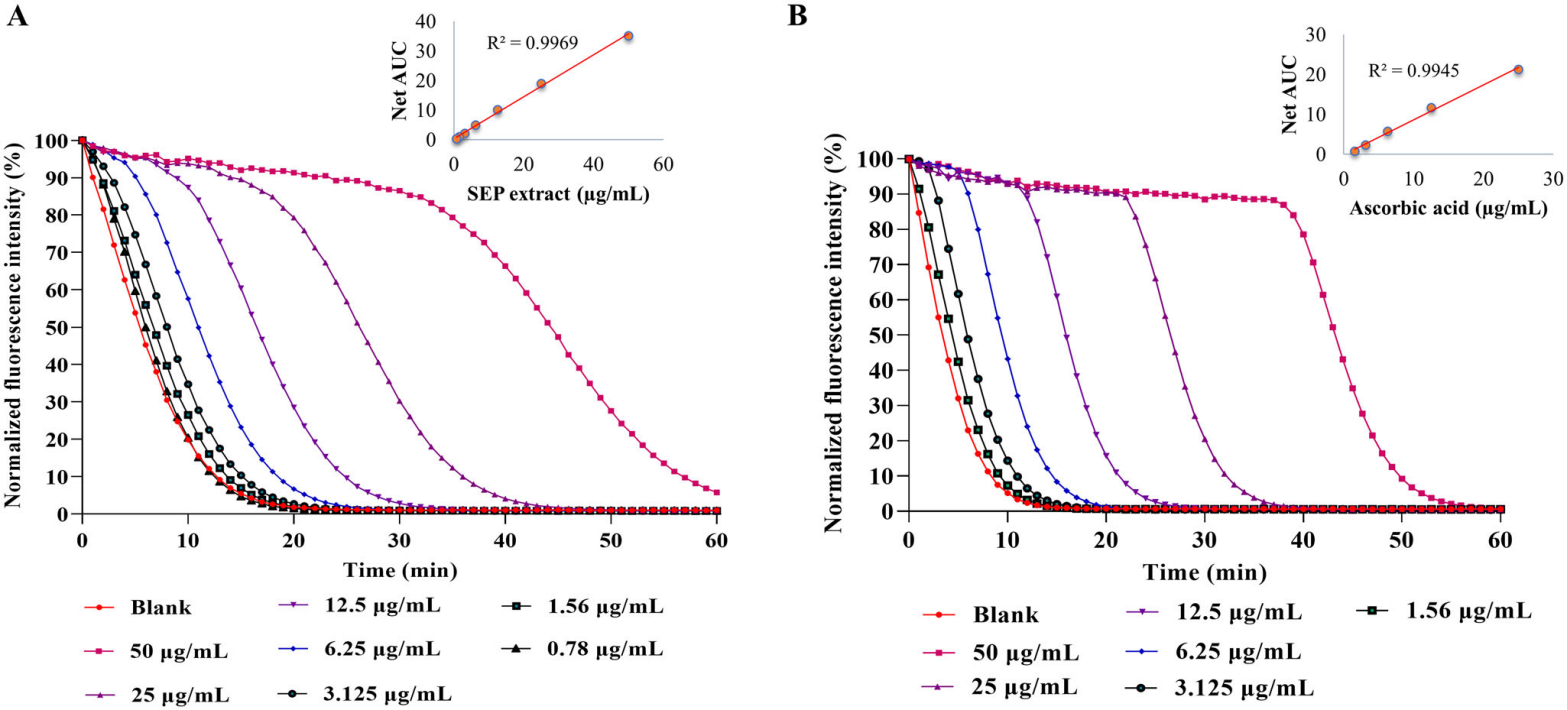
Determination of Oxygen radical absorbance capacity (ORAC) of *E. purpurea* (SEP) extract. ORAC assay was performed for (A) SEP extract and (B) ascorbic acid at respective concentrations and the results expressed as Trolox equivalence/g.

The ORAC assay was used to assess the peroxyl radical scavenging ability of SEP extract (Figure 3(A)). The ORAC value for the extract was found to be 1931.63 µM TE/g. The results were comparable with reference compound ascorbic acid (2378 µM TE/g) (Figure 3(B)).

### Effect of SEP extract on RAW 264.7 cell viability

**Figure 4.**
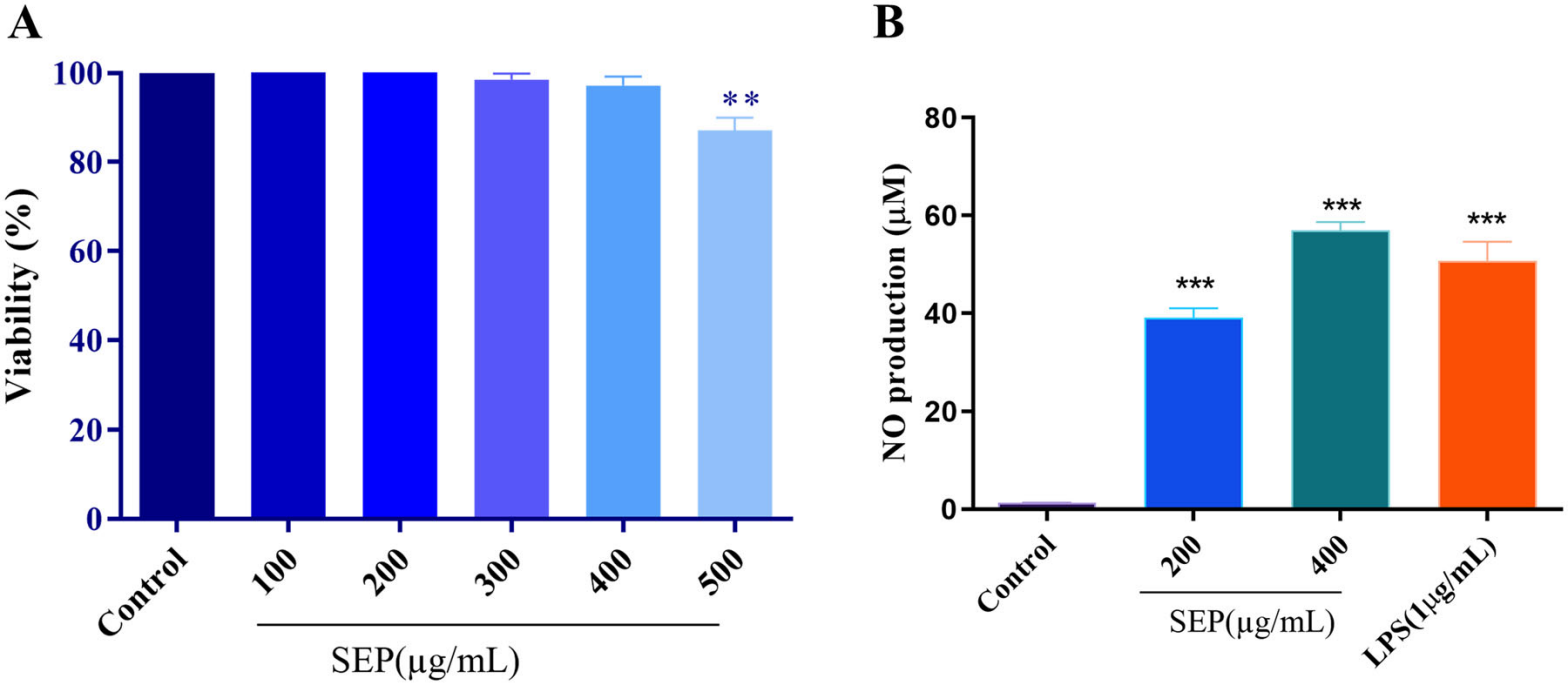
Effect of SEP extract on the viability of RAW264.7 cells and NO production in LPS prompted RAW264.7 cells. (A) The cells were treated with different concentration of SEP extract for 24 h and the cell viability was expressed as the percentage compared with the untreated (control) cell group. (B) The NO level was examined by measuring nitrite level production by Griess reagent and the nitrite production was expressed in micromolar (μM). Data represent mean ± SD of three independent experiments. **p < 0.01 and ***p < 0.001 vs. control.

In the current study, initially we determined the inhibitory effect of SEP extract on the proliferation of the RAW 264.7 cells by using MTT colorimetric assay. As illustrated in Figure 4(A), SEP extract did not show significant cytotoxic effect on the cells up to 400 μg/mL. However, at 500 μg/mL, the SEP extract showed significant decrease in the cell viability (F_(5,12)_ = 8, *p* < 0.001). As a result, we used 200 and 400 μg/mL of SEP extract on RAW 264.7 cells for additional experiments.

### Effect of SEP extract on NO production in macrophages

It has been reported that NO is a major defense molecule generated by activation of macrophages. Treatment with SEP extract on the RAW 264.7 macrophages markedly increased the nitrites production. As shown in Figure 4(B), the normal macrophages generated a tiny amount of NO while it was markedly increased upon SEP extract treatment. At 200 and 400 µg/mL concentrations of SEP extract, the NO release was 30.76-fold and 44.88-fold (*F*_(3,8)_ = 11, *p* < 0.001) higher than the control, respectively. LPS treatment at 1 µg/mL resulted in 39.95-fold increase in NO production compared to control (*F*_(3,8)_ = 11, *p* < 0.001).

### Effect of SEP extract on the phagocytosis of RAW264.7 cells

**Figure 5.**
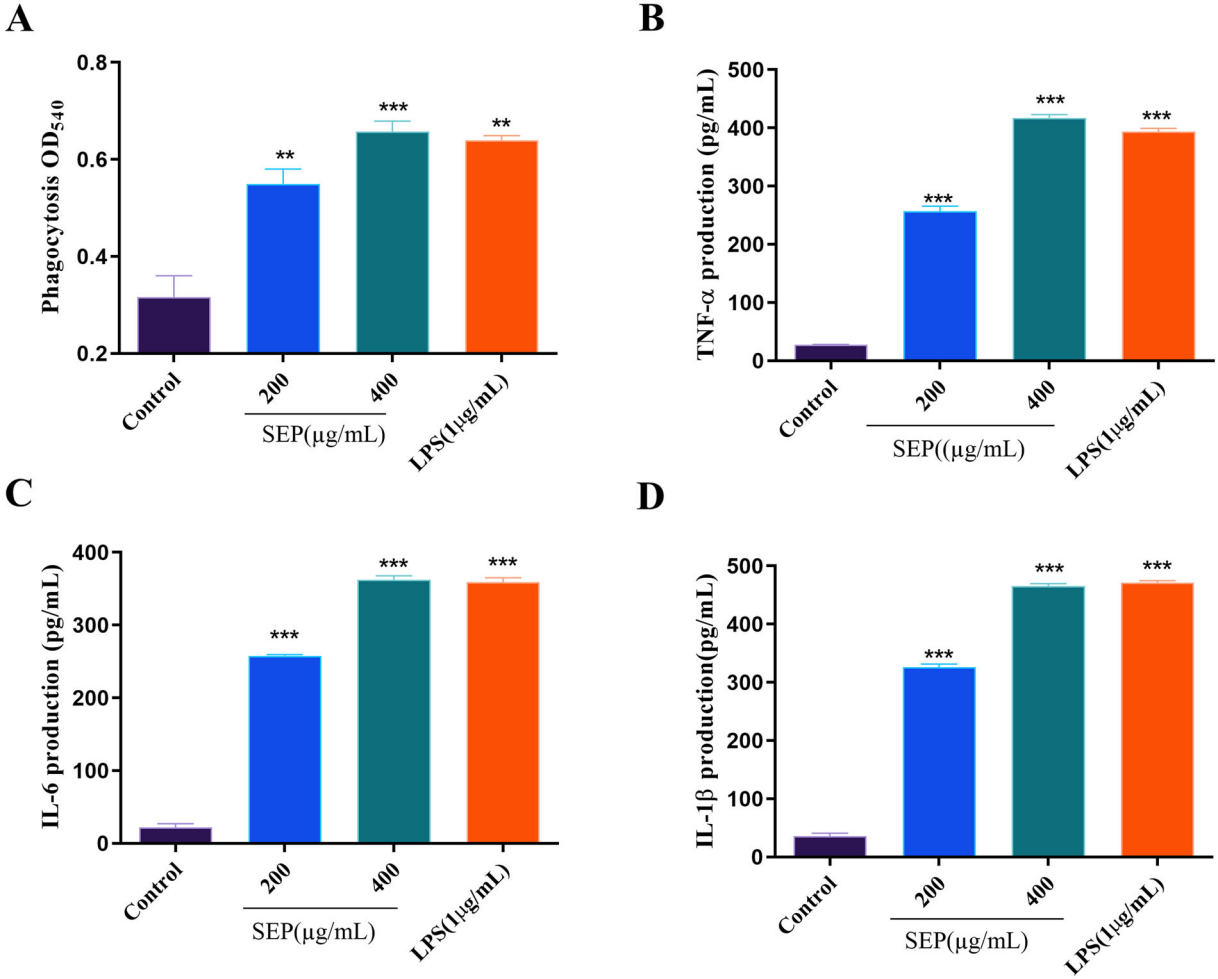
Effect of SEP treatment on RAW 264.7 cells: Phagocytosis (a); secretion levels of TNF-α (B) and IL-6 (C), and IL-1β (D). Data were expressed as the means ± SD (*n* = 3). the group without SEP was used as the negative control, and LPS treatment (5 μg/mL) was used as the positive control group. **p < 0.01 and ***p < 0.001 vs. negative control.

It is well established that macrophages are the most vital phagocytes. The main role of macrophages is to act as armor against invaded pathogens through phagocytosis. Effect of SEP extract on the phagocytosis of RAW264.7 cells is presented in Figure 5(A). Optical density of SEP extract treated macrophage cells significantly rose at 200 (*F*_(3,8)_ = 28.78, *p* < 0.01) and 400 µg/mL (*F*_(3,8)_ = 28.78, *p* < 0.001) compared to normal control cells. At higher dose SEP extract could clearly enhance the level of phagocytosis comparable to LPS treated RAW 264.7 macrophages.

### Effect of SEP extract on the secretion and expression of cytokines in RAW 264.7 macrophages

In the present study, the impact of SEP extract on the release of the inflammatory cytokines from RAW 264.7 cells were analyzed using ELISA. As presented in Figure 5(B,D), the secretion of TNF-α and ILs (IL-6, and IL-1β) were considerably increased in SEP extract-treated cells. At the concentration of 400 µg/mL SEP extract was able to remarkably improve the production of TNF-α by 14.11-fold (*F*_(3,8)_ = 849.6, *p* < 0.001). Similarly, the levels IL-6 and IL-1β production in RAW 264.7 cells treated with SEP extract at the concentration of 400 μg/mL was also increased significantly by 15.33- (*F*_(3,8)_ = 1025, *p* < 0.001) and 12.14-fold (*F*_(3,8)_ = 8, *p* < 0.001), respectively relative to control cells. These results demonstrated the immunomodulatory activities SEP extract in macrophage cells.

### Effect of SEP extract on CTX-induced immunosuppression model in mice

#### Immune organ indices and CTX-induced histopathological changes

**Figure 6.**
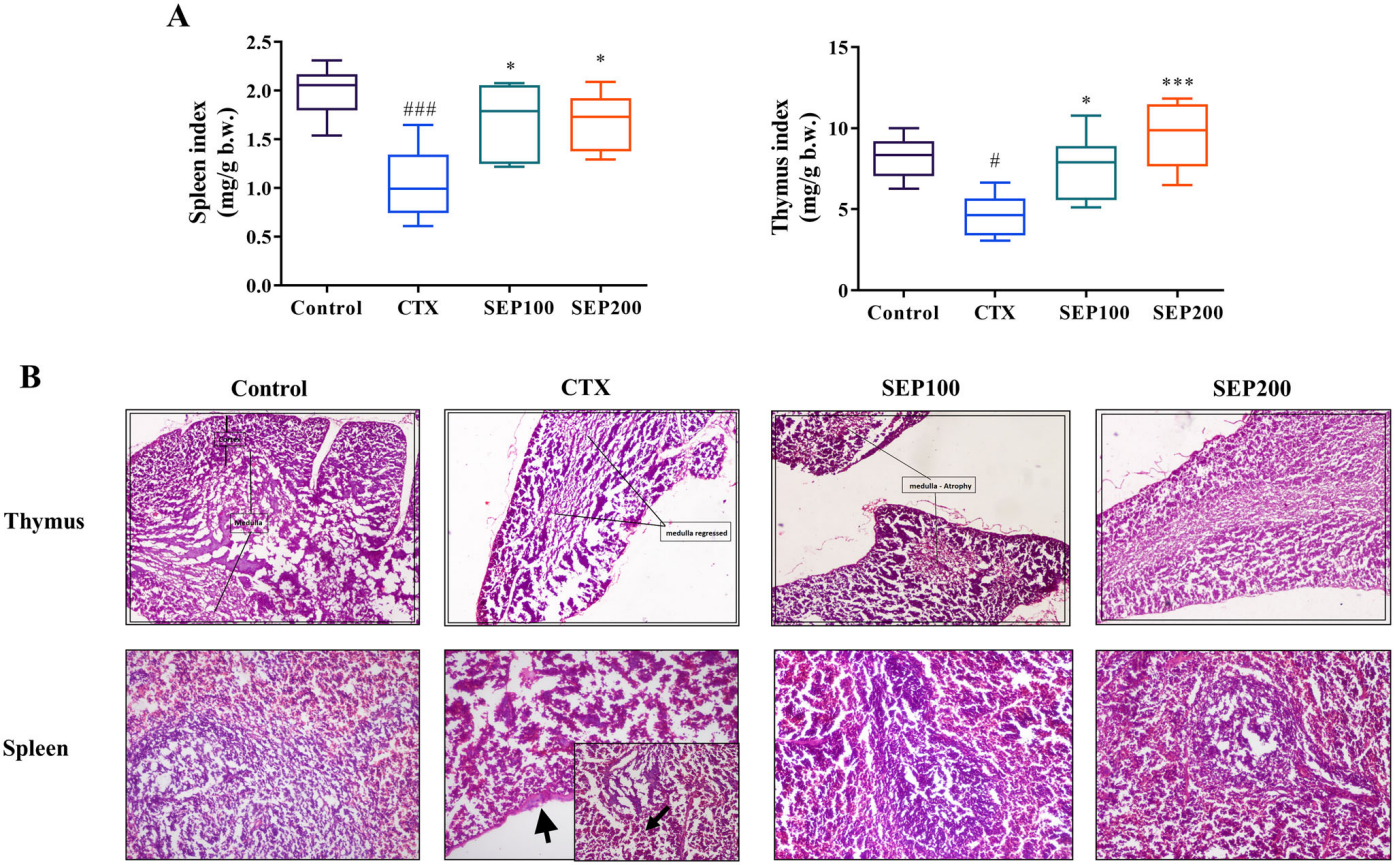
Effect of standardized *E. purpurea* (SEP) extract on immune organs in CTX-treated mice. (A) Organ indices (B) Representative photomicrographs of H&E-stained thymus and spleen for each group (magnification 100×). Control: normal control group; CTX: cyclophosphamide-induced model group; SEP100: challenged with CTX and treated with 100 mg/kg/day SEP extract; SEP200: challenged with CTX and treated with 200 mg/kg/day SEP extract. Data are expressed as mean ± *SD* (*n* = 6). ^#^*p* < 0.05 and ^###^*p* < 0.001 vs. control; *p < 0.05 and ***p < 0.001 vs. CTX group.

There was a normal trend in the body weight gain of control mice from day 0 to 14th day of experiment whereas the model (CTX) group showed noticeable reduction in the body weight throughout the study period compared to control group. Low and high dose SEP extract treatment restored the body weight gain of animals. However, the data were not significant (Data not shown). Figure 6(A) shows the effect of SEP administration on immune organ indices. The CTX group exhibited significant reduction in the spleen (1.91-fold, *F*_(3,20)_ = 8, *p* < 0.001) and thymus (1.77-fold, *F*_(3,20)_ = 8.97, *p* < 0.05) indices compared to the normal control group. SEP treatment groups noticeably improved the organ indices in CTX mice. SEP extract showed 1.62- and 1.61-fold increase in spleen index at 100 and 200 mg/kg doses respectively compared to untreated CTX group (*F*_(3,20)_ = 8, *p* < 0.05). Further, the thymus index was 1.64-(*F*_(3,20)_ = 8.97, *p* < 0.05) and 2.07-fold (*F*_(3,20)_ = 8.97, *p* < 0.001) higher in SEP100 and SEP200 groups respectively, compared to CTX model group.

The effect of SEP extract treatment on immune organs were further assessed by histopathological examination (Figure 6(B)). The H&E staining of thymus demonstrated that the CTX-induced mice showed regressed medullar region with atrophy and fibrosis. Mice in the SEP extract treatment groups, particularly high dose group, showed near normal architecture with increased cortex volume. The CTX-mice showed moderate red pulp hyperplasia, necrotic changes in the white pulp and capsular wall thickening. Compared with CTX group, SEP extract groups showed restoration of spleen architecture with increased white pulp region.

#### Splenic lymphocyte proliferation, NK cell activity and phagocytosis

**Figure 7.**
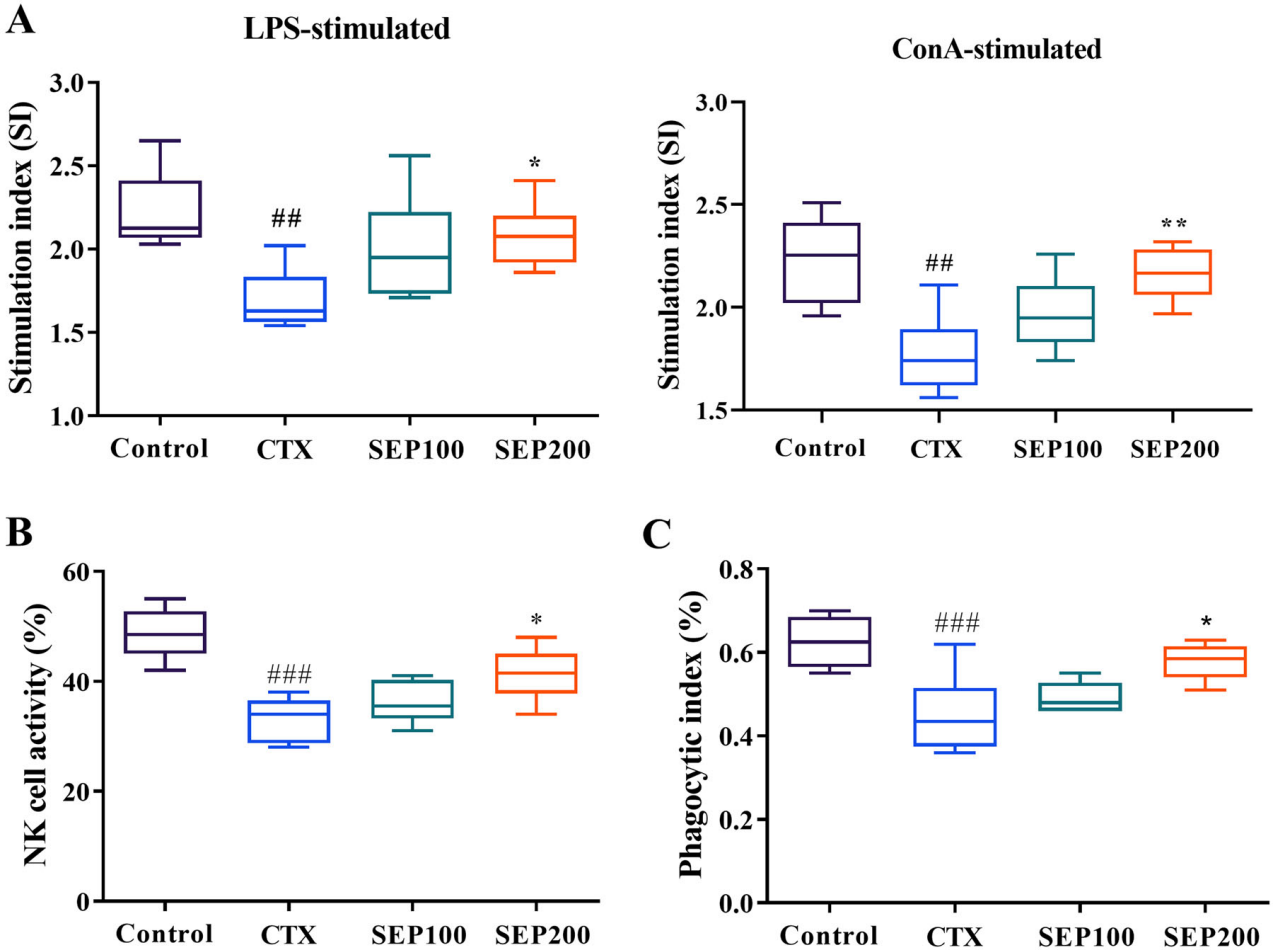
Effect of standardized *E. purpurea* (SEP) extract on immune status in CTX-induced immunosuppression mice. (A) Lipopolysaccharide (LPS)-induced splenocyte proliferation, (B) Natural killer (NK) cell activity and (C) macrophage phagocytosis in CTX model mice. Control: normal control group; CTX: cyclophosphamide-induced model group; SEP100: challenged with CTX and treated with 100 mg/kg/day SEP extract; SEP200: challenged with CTX and treated with 200 mg/kg/day SEP extract. Data are expressed as mean ± *SD* (*n* = 6). ^##^*p* < 0.01 and ^###^*p* < 0.001 vs. control; *p < 0.05 and **p < 0.01 vs. CTX group.

Lymphocyte proliferation is a frequently used parameter to assess the immune response. Figure 7(A,B) shows the effect of SEP extract on the proliferation of LPS and ConA-stimulated splenocytes. The CTX model mice showed an obvious reduction in the LPS-stimulated B-lymphocyte proliferation compared to control group (1.31-fold, *F*_(3,20)_ = 5.40, *p* < 0.01). Further, the T-lymphocyte proliferation was significantly decreased in CTX group compared to normal control group (*F*_(3,20)_ = 8.02, 1.26-fold, *p* < 0.01). The splenic proliferation was however improved dose dependently in SEP extract treatment groups. There was a significant increase in the stimulation index of LPS stimulated splenocytes in the SEP200 group as compared to the untreated CTX mice (*F*_(3,20)_ = 5.40, *p* < 0.05). Similar trend was observed in ConA-stimulated splenocyte proliferation where stimulation index was markedly higher in the SEP200 group compared to the CTX group (*F*_(3,20)_ = 8.02, *p* < 0.01).

NK cells are the cytotoxic immune cells, the activity of which reflects the immune response to a greater extent. In this study, the CTX model mice exhibited significantly lower NK cell toxicity as compared to normal mice (*F*_(3,20)_ = 15.17, *p* < 0.001). SEP extract treatment at 100 and 200 mg/kg doses showed 9.04% and 24.6% (*F*_(3,20)_ = 15.17, *p* < 0.05) increase in the NK cell activity compared to CTX group (Figure 7(C)).

The macrophage phagocytosis is another crucial measure of the immune function. Figure 7(D) shows the effect of SEP extract on the phagocytic index of peritoneal macrophages. CTX group showed 27.73% reduction in the phagocytic index compared to control group (*F*_(3,20)_ = 9, *p* < 0.001). SEP extract could increase the phagocytic index by 8.86% and 28.03% (*F*_(3,20)_ = 9, *p* < 0.05) at 100 mg and 200 mg/kg doses respectively as compared to the untreated CTX group

#### Serum levels of inflammatory markers in immunosuppressed mice

**Figure 8.**
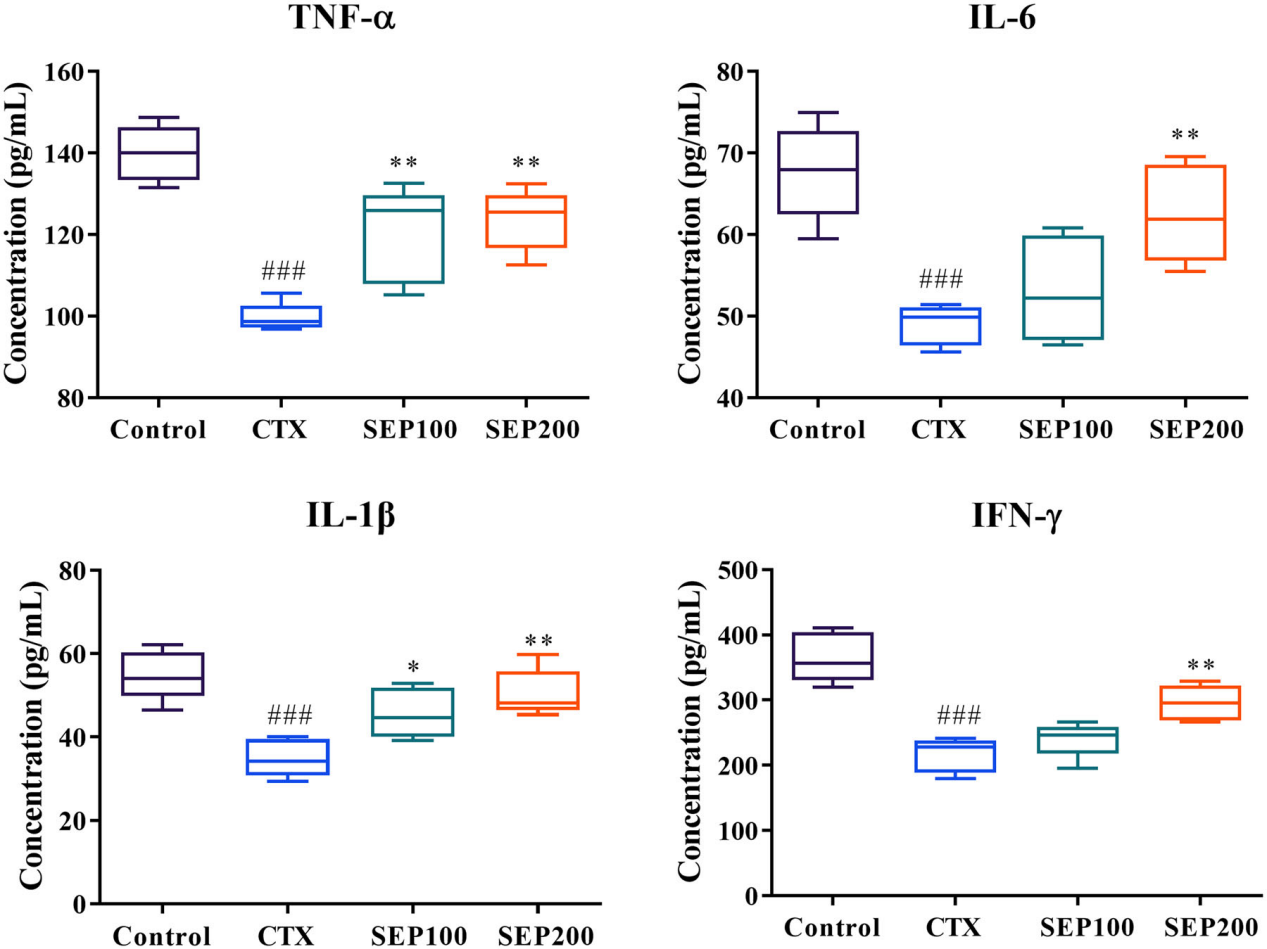
Effect of standardized *E. purpurea* (SEP) extract administration on serum cytokine levels of CTX model mice. Control: normal control group; CTX: cyclophosphamide-induced model group; SEP100: challenged with CTX and treated with 100 mg/kg/day SEP extract; SEP200: challenged with CTX and treated with 200 mg/kg/day SEP extract. Data are expressed as mean ± *SD* (*n* = 6). ^###^*p* < 0.001 vs. control; *p < 0.05 and **p < 0.01 vs. CTX group.

Figure 8 shows the effect of SEP extract on serum levels of inflammatory cytokines in mice. The CTX group showed a significant reduction in the serum levels of TNF-α (1.4-fold, *F*_(3,16)_ = 21.80, *p* < 0.001), IL-6 (1.38-fold, *F*_(3,16)_ = 12.42, *p* < 0.001), IL-1β (1.57-fold, *F*_(3,16)_ = 11.95, *p* < 0.001) and IFN-γ (1.69-fold, *F*_(3,16)_ = 24.28, *p* < 0.001), compared to normal control group. However, SEP extract treatment markedly restored the levels of these cytokines in CTX-induced mice. Low dose (100 mg/kg) of SEP extract could significantly increase the TNF-α (1.21-fold, *F*_(3,16)_ = 21.80, *p* < 0.01) and IL-1β (1.31-fold, *F*_(3,16)_ = 11.95, *p* < 0.05) levels compared to CTX group while the levels of IL-6 and IFN-γ were insignificantly altered. The SEP200 group exhibited profound immunomodulatory activity as evident from the significantly higher levels of the cytokines in comparison with CTX group (*p* < 0.01).

## Discussion

Preparations from *Echinacea* have been widely implicated in several physiological benefits such as anti-inflammatory, immunomodulatory, antiviral and antioxidant properties (Sharma et al. 2006; Ramasahayam et al. 2011). Chicoric acid, a major active principle in *E. purpurea* extract is documented as a chemical marker to ascertain the quality of preparations from the plant (Zhang et al. 2011). The present study reports for the first time the immunomodulatory functions of a standardized extract from *E. purpurea* arial parts containing higher content (not less than 4%) of chicoric acid. In the preliminary screening, the extract showed noticeable radical scavenging activity possibly due to the presence of chicoric acid and other polyphenols (Ramezannezhad et al. 2019).

Several studies have showed that activated macrophages can execute a series of biological events, such as phagocytosis, chemotaxis, and destruction of intended organisms, to scavenge external substances and infectious microbes all through the innate immune response (Hirayama et al. 2017). Hence, we have investigated the immunomodulatory effects of SEP extract at non-cytotoxic concentrations initially in RAW 264.7 murine macrophage cells. Further, CTX-induced immunosuppression mouse model was used to evaluate the claims *in vivo*.

NO acts as an immunoregulatory mediator that participates in both innate and adaptive immune responses (Galeas-Pena et al. 2019). Previously it is reported that NO releasing enhances the immunomodulatory function by upregulating the iNOS expression at the protein and gene levels (Kumar et al. 2022). In the present study, a considerable release of NO from RAW 264.7 cells exposed to SEP extract was evident compared to untreated cells. These data were highly encouraging to investigate further on the immune boosting potentials of SEP extract.

Phagocytic activity is one of the most distinctive characteristics in the activated macrophages (Wang et al. 2016). In the present study SEP extract markedly improved the phagocytosis of macrophages *in vitro*. Consistently the extract showed significantly higher phagocytic index at 200 mg/kg b.w. dose as compared to untreated immunosuppression mice. These results suggest that SEP extract may effectively scavenge foreign invasion and enhance the immunity (Fan et al. 2015; Gordon 2016).

Cytokines are crucial in regulating the immune response, mediate inflammation and involve in cell differentiation of immune system (Dirchwolf et al. 2016). TNF-α and IL-6 are the key cytokines produced by the immune cells as inflammatory response (Jacques et al. 2009). In the present study, SEP extract stimulated the RAW 264.7 cells to release TNF-α, IL-6 and IL-1β. It was further confirmed in the immunosuppressed mice that the administration of SEP extract could substantially stimulate immunogenic response by the cytokine released into the systemic circulation.

It is important to assess the lymphocyte proliferation to study the adaptive immunity of animals (Singh et al. 2016). In the present study, we found that SEP extract at 200 mg/kg b.w. could significantly improve the ConA and LPS stimulated T and B lymphocyte proliferation respectively in immunosuppressed mice. In addition, the SEP extract triggered the NK cell cytotoxicity which further support the claim that SEP extract can stimulate adaptive immune response.

Nevertheless, there are reports on the immunomodulatory effect of *E. purpurea* extracts, the present study rationalized the higher content of chicoric acid in the standardized extract which could substantially contribute to its efficacy alongside other polyphenols and polysaccharides. Caffeic acid derivatives exert immunomodulatory activity and ameliorate inflammation (Ardjomand-Woelkart and Bauer 2016; Cao et al. 2019). Chicoric acid, the caffeic acid derivative present predominantly in *E. purpurea* has been studied for various biological functions including immune regulatory and anti-inflammatory activities (Kour and Bani 2011; Tsai et al. 2017). Recently, Wang et al. (2020) reported that chicoric acid extracted from *E. purpurea* could significantly regulate the immunity of yaks by enhancing the proliferation of peripheral blood mononuclear cells. In the light of these reports and the experimental data of the present investigation, the authors are of the opinion that a standardized *E. purpurea* extract with not less than 4% chicoric acid can be better explored as immune booster in immunosuppressed individuals.

## Conclusion

In this study, SEP extract with 4% chicoric acid could significantly stimulate the immune response in Raw 264.7 cells, at 200 and 400 µg/mL concentrations. Further, SEP extract at 200 mg/kg dose could substantially improve the T-and B-lymphocyte proliferation, NK cell activity, and macrophage phagocytosis in immunosuppressed mice. Together, the present study illustrates that SEP extract with standardized content of chicoric acid can be used as a functional ingredient for immunomodulation.

## Data Availability

The data sets used and/or analyzed during the current study available from the corresponding author on reasonable request.
